# The Immunogenicity of Branded and Biosimilar Infliximab in Rheumatoid Arthritis According to Th9-Related Responses

**DOI:** 10.3390/ijms18102127

**Published:** 2017-10-12

**Authors:** Rossella Talotta, Angela Berzi, Andrea Doria, Alberto Batticciotto, Maria Chiara Ditto, Fabiola Atzeni, Piercarlo Sarzi-Puttini, Daria Trabattoni

**Affiliations:** 1 Department of Rheumatology, Azienda Ospedaliera-Polo Universitario Luigi Sacco, Milan 20157, Italy; alberto.batticciotto@hsacco.it (A.B.); mariachiara.ditto@hsacco.it (M.C.D.); atzenifabiola@gmail.com (F.A.); sarzi@tiscali.it (P.S.-P.); 2 Department of Biomedical and Clinical Sciences, Azienda Ospedaliera-Polo Universitario Luigi Sacco, Milan 20157, Italy; angyberzi@gmail.com (A.B.); daria.trabattoni@unimi.it (D.T.); 3 Department of Rheumatology, University of Padua, Padua 35100, Italy; adoria@unipd.it

**Keywords:** biosimilars, Th9 lymphocytes, rheumatoid arthritis, infliximab

## Abstract

Our objective was to evaluate the immunogenicity of branded and biosimilar infliximab by detecting changes in T-helper-9 (Th9) percentages induced by an in vitro stimulation test. Methods: Peripheral blood mononuclear cells collected from 55 consecutive rheumatoid arthritis (RA) outpatients (15 drug free, 20 successfully treated with branded infliximab, 20 branded infliximab inadequate responders) and 10 healthy controls were cultured, with or without 50 μg/mL of infliximab originator (Remicade^®^) or 50 μg/mL of infliximab biosimilar (Remsima^®^) for 18 h. Th9 lymphocytes were identified by means of flow cytometry as PU.1 and IRF4-expressing, IL-9-secreting CD4^+^ T cells. Furthermore, the markers CCR7 and CD45RA were used to distinguish naïve from memory IL-9 producer cells. Results: Under unstimulated conditions, the drug-free RA patients had the highest percentages of Th9 lymphocytes. Following stimulation with branded infliximab, the percentages of PU.1 and IRF4-expressing Th9 cells, CCR7^+^, CD45RA^−^ (central memory) and CCR7^−^, CD45RA^−^ (effector memory) cells significantly increased in the group of inadequate responders, but no significant variation was observed after exposure to the biosimilar of infliximab. Conclusions: Th9 cells seem to be involved in the immune response to the epitopes of branded, but not biosimilar, infliximab, and this may depend on the recall and stimulation of both central and effector memory cells.

## 1. Introduction

Rheumatoid arthritis (RA) is an autoimmune chronic disease characterized by inflammation of peripheral joints, with a varying degree of systemic involvement. The pathogenesis is partly understood and relies on the activation of cells belonging either to innate and adaptive immunity, with the subsequent production of cytokines and chemokines contributing to final synovitis and systemic inflammation. The treatment of RA has been remarkably implemented in recent decades. Several conventional and biological drugs have been developed in order to counteract the activation of the immune system by acting at different steps of the inflammatory cascade. Particularly, biological agents currently approved for RA include: anti-Tumor Necrosis Factor-α (TNF) drugs (infliximab, etanercept, adalimumab, certolizumab pegol, golimumab), an anti-CD20 drug (rituximab), a receptor antagonist of interleukin-1 (anakinra), an antagonist of the receptor of interleukin-6 (tocilizumab) and a fusion protein containing the Cytotoxic T Lymphocyte Antigen-4 domain activity (abatacept). All these drugs are characterized by high specificity that allows the recognition of a specific molecule, thus preventing further repercussions on other cells or organs. In addition, biosimilar drugs of infliximab, etanercept and rituximab have been recently commercialised, and their use has been spread due to their non-negligible cost-sparing effects and comparable profiles, in terms of efficacy and safety, with the reference products.

The use of biological drugs to treat RA has led to considerable improvements in inflammation control and the prevention of structural damage. However, there are patients who develop adverse events or experience the progressive loss of treatment efficacy. The unsuccessful outcome of biological therapy may be due to immunogenicity (i.e., the capacity of a drug to induce an immune-mediated response against its own epitopes), which may depend on a number of drug- and patient-related factors. Biological monoclonal antibodies (MoAbs) are synthesised in murine cells and contain some foreign amino acid sequences that are potentially highly antigenic. The 25% murine structure of infliximab and its biosimilar compounds make them more immunogenic than other anti-rheumatic biological agents, despite comparable immunogenicity profiles, as assessed by the production of anti-drug antibodies (ADAs) between originators and biosimilars.

RA is characterized by an aberrant activation of adaptive immunity that is mirrored by the interplay of many sub-sets of T helper (Th) lymphocytes. In an altered cytokine background, such as that of RA patients, the administration of biological drugs that are potentially highly antigenic may induce the aberrant activation of specific T and B effector responses against drug epitopes. There is considerable evidence that the production of ADAs mainly depends on the activation of a Th2 cell pathway; however, as suggested by a few reports, the paradoxical activation of Th1 and Th17 responses following the administration of infliximab may also occur in non-responding RA patients.

The levels of Th9 cells, which are specialised for producing IL-9, but can also produce IL-10, IL-17, IL-21 and IL-22, are increased in the bloodstream and synovial membranes of RA patients, where they are directly related to the degree of lymphoid organisation and the production of autoantibodies, such as anti-citrullinated peptide antibodies (ACPAs). However, no study has yet investigated the role of Th9 lymphocytes in the immunogenicity of biological agents by comparing originator and biosimilar compounds.

The aim of this study was to evaluate if Th-9 cells can mediate drug immunogenicity and to compare the Th9-related immunogenicity of the infliximab originator (Remicade^®^) and its biosimilar compound (Remsima^®^) in a cohort of infliximab-responder and inadequate responder (IR) RA patients by means of an in vitro stimulation assay, taking into account the demographic and clinimetric features of the patients, the use of concomitant drugs, and the reason for discontinuing infliximab.

## 2. Results

### 2.1. Baseline Demographic and Clinical Assessment

At the time of enrolment, five of the 15 drug-naïve RA patients (14 Caucasians and one Chinese; 12 females; mean age 54.8 ± 16.2 years; mean disease duration 2.3 ± 3.9 years) had ACPAs, seven had rheumatoid factor (RF), and two had anti-nuclear antibodies (ANAs); their mean C-reactive protein/28-joint disease activity score (CRP-DAS28) was 4.6 ± 1.0. All were taking anti-inflammatory and analgesic drugs as needed.

Fifteen of the 20 good responders to infliximab (19 Caucasians and one Hispanic; 16 females, mean age 61.3 ± 12.2 years; mean disease duration 13.4 ± 7.2 years) had ACPAs, 12 had ANAs, 11 had RF, three had anti-double stranded DNA antibodies (anti-dsDNA), one had anticardiolipin antibodies (ACLAs), and one had anti-extractable nuclear antigen antibodies (ENAs). Their RA had been well controlled by infliximab (Remicade^®^) for a mean of 8.3 ± 3.9 years (mean CRP-DAS28 at the time of blood sampling 2.5 ± 1.0). The concomitant medications were prednisone (2.5–10 mg/day) in eight patients, methotrexate (5–15 mg/week) in 20, and hydroxychloroquine (200–400 mg/day) in three.

Seventeen of the 20 non-responders to infliximab (19 Caucasians and one Indian; 15 females; mean age 57.0 ± 12.2 years; mean disease duration 18.1 ± 9.5 years) had ANAs, 15 had ACPAs, 15 had RF, three had ACLAs, two had anti-dsDNA, and two had anti-ENAs. Thirteen were being treated with intravenous (i.v.) abatacept (10 mg/kg every four weeks), five were being treated with i.v. tocilizumab (8 mg/kg every four weeks), one was being treated with subcutaneous (s.c.) etanercept (50 mg once a week), and one was being treated with s.c. certolizumab pegol (200 mg every other week); these treatments were the second (8 patients), third (8 patients) or fourth biological line (4 patients). The patients had been treated with infliximab (Remicade^®^) for a mean of 2.4 ± 1.9 years, and had discontinued the drug for a mean of 8.0 ± 2.5 years due to inefficacy (11 cases) or adverse events (mainly allergic or infusion reactions, 9 cases). Their concomitant conventional drugs were prednisone (2.5–10 mg/day) in 14 cases, methotrexate (5–15 mg/week) in nine cases and hydroxychloroquine (200–400 mg/day) in five cases. Their mean CRP-DAS28 was 2.9 ± 0.8 at the time of blood sampling. Demographic and clinical characteristics are displayed in Table 1.

Treated patients and untreated patients were matched for gender and age, and significantly differed for disease duration (*p* < 0.001, Student’s *t* Test for unpaired samples); ANAs and ACPAs were more frequently detected in longstanding RA treated patients than in untreated ones (*p* < 0.001 and *p* = 0.006, respectively; Pearson’s Chi squared test).

Good responders and non-responders to infliximab were matched for gender, age, disease duration, autoantibody subsets (Student’s *t* Test for unpaired samples and Pearson’s Chi squared test); whereas they significantly differed for methotrexate and prednisone medium dose intake, (respectively *p* = 0.003 and 0.030; Student’s *t* Test for unpaired samples).

### 2.2. T helper 9 Cells at Baseline

**Figure 1:**
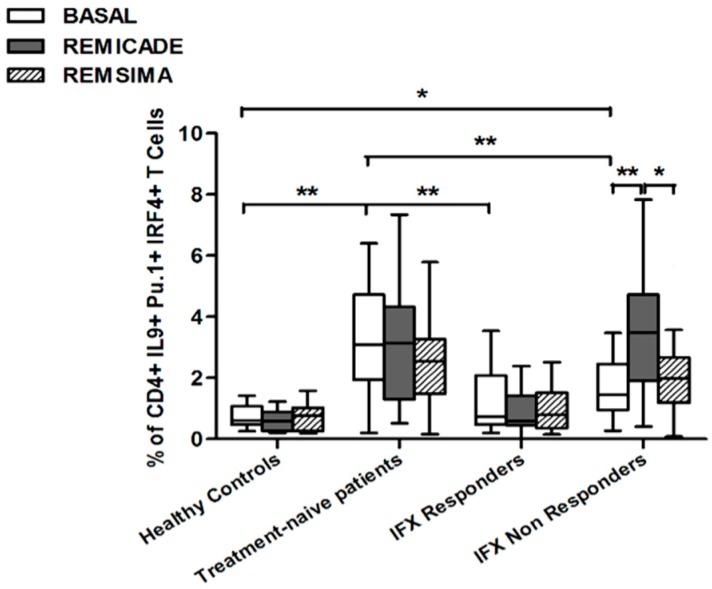
Percentages of PU.1^+^, IRF4^+^, IL-9^+^ CD4^+^ T cells at baseline and after exposure to branded and biosimilar infliximab. * *p* < 0.05, ** *p* < 0.01.

**Figure 2:**
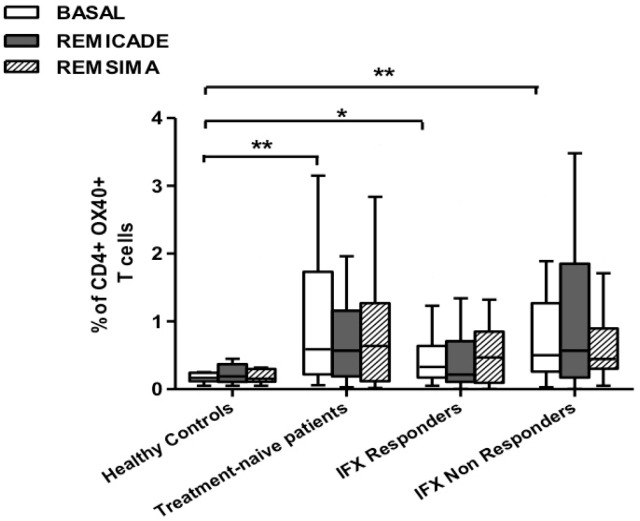
Percentages of OX40^+^, IL-9^+^ CD4^+^ T cells at baseline and after exposure to branded and biosimilar infliximab. * *p* < 0.05, ** *p* <0.01.

The baseline percentage of PU.1^+^, IRF4^+^ Th9 cells was higher in the drug-naïve patients than in the healthy controls and treated patients (*p* < 0.01) (Figure 1). There was no significant difference in the percentage of OX40-expressing, IL-9-producing, CD4^+^ T cells between the healthy controls and any of the patient groups (Figure 2), possibly because of the involvement of different pathways in the differentiation of Th9 cells [1]; however, the percentage of OX40-expressing CD4^+^ T cells was higher in the patient groups than in the controls. The greater frequency of Th9 cells among the RA patients was not associated with higher ANA or other autoantibody levels, disease duration, baseline CRP-DAS28, nor was it associated with the reason for discontinuing infliximab or the number of previous biological drugs administered to the non-responders. Moreover, a multivariate analysis did not reveal any significant influence of concomitant conventional or biological treatments, although the heterogeneity of the biological therapies and the limited number of cases may have biased the statistical evaluation.

In brief, at baseline the difference in the percentage of Th9 cells between the healthy controls and the RA patients was observed in the group of untreated patients. This finding indicates that the activation of Th9 cells is a distinctive characteristic of RA and can be restored by concomitant efficacious conventional or biological treatments.

### 2.3. Effects of Infliximab (Remicade^®^) on T Helper 9 Cells

Stimulation with branded infliximab increased the percentage of PU.1^+^ and IRF4^+^ Th9 cells only in the IR group of patients (Figure 1). There were no differences in OX40-expressing, IL-9-producing CD4^+^ T cells or OX40-expressing CD4^+^ T cells, before and after infliximab exposure (Figure 2), possibly because of the widespread expression of OX40 in the Th cell pool [2].

**Figure 3:**
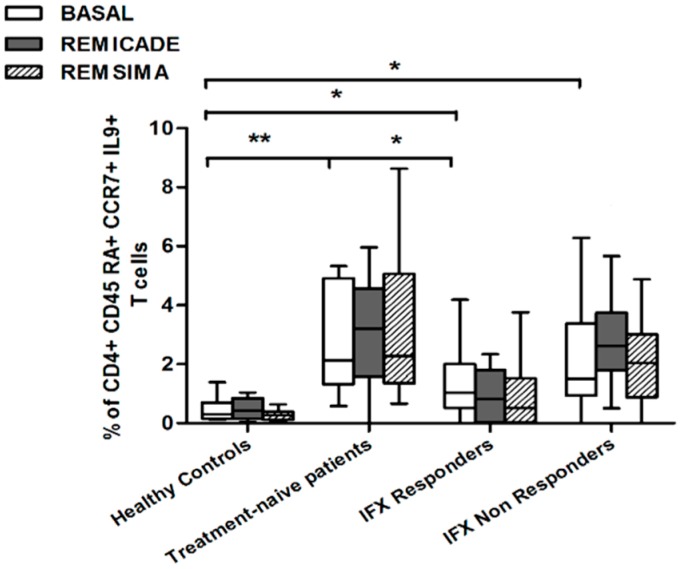
Percentages of CD45RA^+^, CCR7^+^, IL-9^+^ CD4^+^ (naïve) T cells at baseline and after exposure to branded and biosimilar infliximab. * *p* < 0.05, ** *p* <0.01.

**Figure 4:**
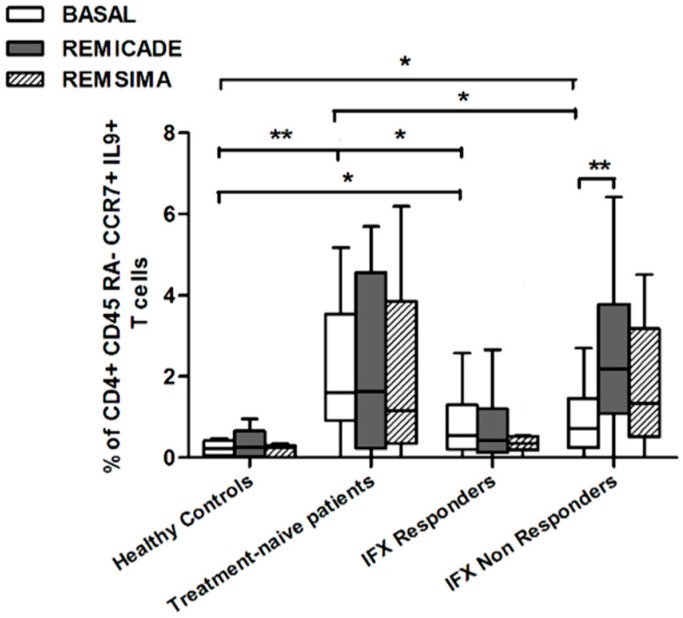
Percentages of CD45RA^−^, CCR7^+^, IL-9^+^ CD4^+^ (central memory) T cells at baseline and after exposure to branded and biosimilar infliximab. * *p* < 0.05, ** *p* <0.01.

**Figure 5:**
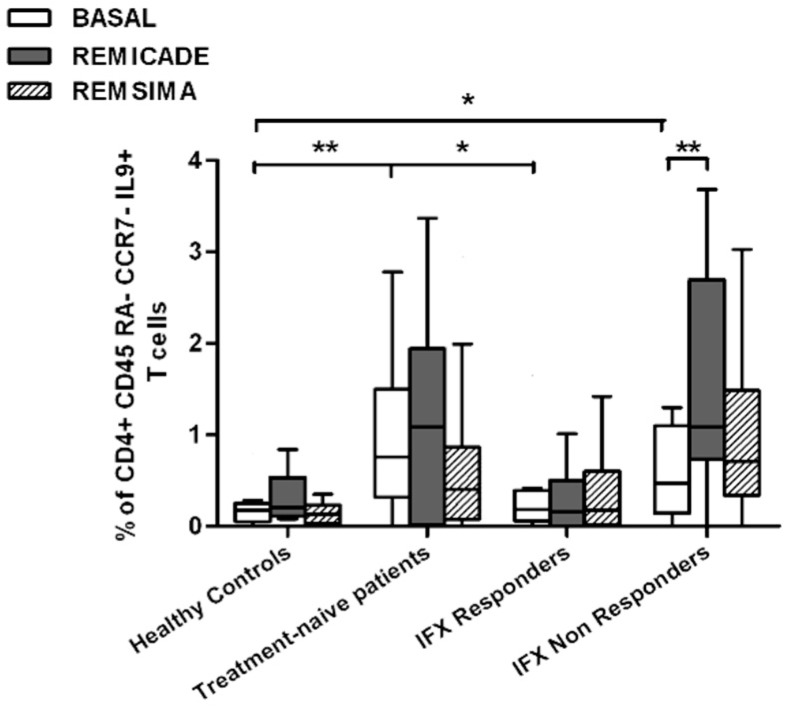
Percentages of CD45RA^−^, CCR7^−^, IL-9^+^ CD4^+^ (effector memory) T cells at baseline and after exposure to branded and biosimilar infliximab. * *p* < 0.05, ** *p* <0.01.

**Figure 6:**
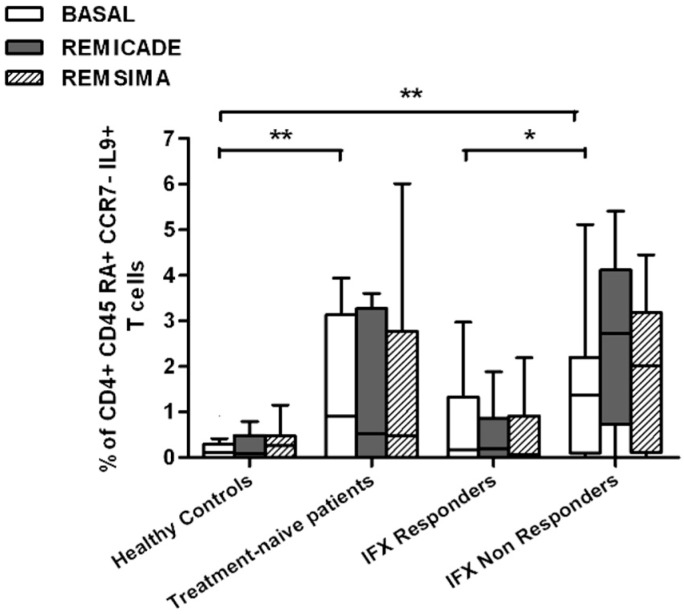
Percentages of CD45RA^+^, CCR7^−^, IL-9^+^ CD4^+^ (terminally differentiated effector memory, TEMRA) T cells at baseline and after exposure to branded and biosimilar infliximab. * *p* < 0.05, ** *p* <0.01.

We also investigated whether Th9 lymphocytes may be activated by means of a specific stimulus on Th memory cells from patients who had discontinued infliximab because of inefficacy or adverse events. Antigen stimulation can induce central memory (CCR7^+^, CD45RA^−^) T cells to migrate from lymph nodes to peripheral tissues, lose CCR7, and differentiate into (CCR7^−^, CD45RA^−^) effector memory T cells with immediate activation. Furthermore, in the case of protracted low-dose antigen stimulation, they may be able to re-express the molecule CD45RA (terminally differentiated effector memory, TEMRA) and acquire surveillance functions with less pronounced effector properties [3,4]. We therefore subdivided IL-9-secreting CD4^+^ T cells on the basis of the expression of CCR7 and CD45RA, which makes it possible to distinguish among naïve, central memory, effector memory and TEMRA cells. All of these cell pools were increased in the untreated RA patients in comparison with the other groups. Following the addition of infliximab, IL-9^+^, CCR7^+^, CD45RA^−^ central memory cells and IL-9^+^, CCR7^−^, CD45RA^–^ effector memory cells (but not naïve Th9 cells) were increased in the infliximab IR group, thus indicating that these cell pools may account for the change in the percentage in PU.1 and IRF4-expressing, IL-9-secreting CD4^+^ T cells (Figure 3, Figure 4 and Figure 5). On the contrary, the percentage of TEMRA lymphocytes did not vary, presumably because of the limited proliferative activity of this cell pool (Figure 6).

**Figure 7:**
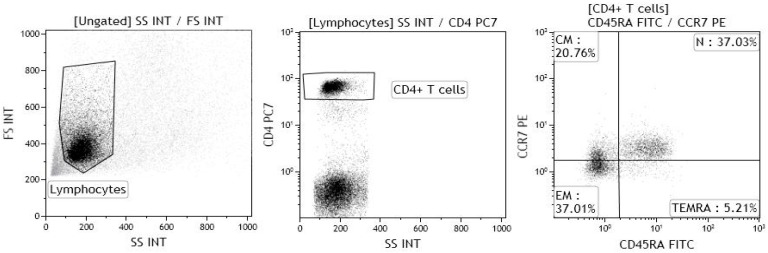
Gating strategy for the identification of naïve CD4^+^ T lymphocytes (N), central memory CD4^+^ T lymphocytes (CM), effector memory CD4^+^ T lymphocytes (EM) and terminally differentiated effector memory (TEMRA) CD4^+^ T lymphocytes. The lymphocyte population was gated on forward and side scatter properties, and further gated for CD4, CCR7, CD45RA expression; at least 20,000 events were acquired within the CD4 gate. The samples were acquired using a Gallios flow cytometer, and the data were analysed using Kaluza software (both Beckman Coulter).

The gating strategy for the identification of naïve, central memory, effector memory and TEMRA CD4^+^ T lymphocytes is shown in Figure 7.

### 2.4. Comparison of the Effects of Remicade^®^ and Remsima^®^ on T Helper 9 Cells

We repeated the previous experiment using the biosimilar compound CT-P13 (Remsima^®^). As in the experiment with the original infliximab, there were no significant variations in the percentages of PU.1^+^, IRF4^+^ Th9 cells, under basal or stimulated conditions, in the healthy controls and the untreated or responding RA patients, but the addition of the biosimilar did not significantly increase the percentage of PU.1 and IRF4-expressing, IL-9-secreting CD4^+^ T cells or IL-9-secreting central and effector memory CD4^+^ T cells in the infliximab IR group, as was observed after the addition of branded infliximab (Figure 1, Figure 4 and Figure 5). Furthermore, as in the case of branded infliximab, exposure to biosimilar CT-P13 did not induce a significant change in the percentages of IL-9-secreting naïve and TEMRA CD4^+^ T lymphocytes (Figure 3 and Figure 6), OX40-expressing, IL-9-producing CD4^+^ T cells or OX40-expressing CD4^+^ T cells in any of the patient groups (Figure 2).

## 3. Discussion

The aims of this study were to investigate the possible relationship between Th9 cells and the outcome of infliximab biological therapy, and to demonstrate that the immunogenic profiles of branded and biosimilar infliximab are comparable in terms of Th9-driven immune responses. The immunogenicity of a drug depends on the presence of the specific B and T epitopes contained in the primary amino acid sequence, or developing during post-translational modifications [5,6,7]. The most widely accepted hypothesis is that the production of ADAs is the mechanism underlying a drug-induced immune response [8,9,10,11,12], although it may also be responsible for the development of adverse events or a progressive loss of efficacy. The immunogenicity of biological drugs (especially chimeric molecules such as infliximab) may be partly due to the induction of ADAs, but we and other authors have shown that other immune pathways, such as the antigen-specific activation of Th1 or Th17 cells may be an alternative explanation for the rejection of biological treatments [13,14]. Th9 cells are a sub-set of T helper cells that develop from naïve or primed Th2 lymphocytes in the presence of IL-4 and TGFβ, and are characterised by transcriptional factors—PU-1 and IRF4. Furthermore, co-stimulatory molecules (including OX40 and Notch) and other cytokines (such as IL-1β, IL-25, IL-33, type I interferons, and thymic stromal lymphopoietin) are involved in promoting Th9 differentiation and IL-9 production [1]. These cells represent the main source of IL-9, although other cells such as Th2, Th17 and Treg cells may also make a contribution [15,16]. IL-9 is capable of activating various cells, including Th17 and Treg lymphocytes [17], and therefore, depending on the local microenvironment, Th9 lymphocytes may direct the immune response towards autoimmunity/inflammation or tolerance. IL-9 and Th9 cells are increased in subjects with inflammatory arthritis, connective tissue diseases, autoimmune colitis, and autoimmune encephalomyelitis [18,19,20,21,22,23,24]. In a previous experiment, we found an increased prevalence of IFNγ-, IL-4-, IL-17-, IL-9-secreting CD4^+^ T cells in RA patients, although there was no significant association with therapeutic outcomes [25]; this unusual behaviour may be related to the heterogeneity and plasticity of IL-9-producing CD4^+^ T cells, which may also include regulatory cells [26].

In line with these data, our results showed a higher percentage of Th9 cells in the peripheral blood of RA patients than in that of healthy controls; the levels were particularly high in untreated patients, whereas treatment with conventional or biological drugs seemed to reduce the difference. Following stimulation with original infliximab, the percentage of PU.1^+^, IRF4^+^ Th9 cells increased in the infliximab IR group and this finding was confirmed when the experiment was repeated with central and effector memory IL-9^+^, CD4^+^ T cells, but not with naïve or TEMRA IL-9 producer CD4^+^ T cells.

PU1^+^, IRF4^+^ Th9 cells may increase following antigenic stimulation with infliximab in patients who have discontinued treatment, possibly because of the recall and activation of central and effector memory Th9 cells, but when we assessed the response of PU.1 and IRF4-expressing, IL-9-secreting CD4^+^ T lymphocytes to biosimilar infliximab, we did not find any significant variation from baseline in any of the four groups, although there was a trend in the case of central and effector memory cells. A possible inhibitory effect on memory Th cells related to IL-10, produced in vitro upon reverse signaling on mTNF-α-bearing dendritic cells (DCs), or by memory T cells producing IL-10—particularly expanded in tolerant patients—cannot be excluded. Effector memory Th cells were reported to be consistently blocked by the IL-10, induced in vitro by the drug in tolerant patients and, to a lesser extent, in patients who interrupted therapy [27]. In our previous study [13], we reported an increase in the percentage of Treg cells in IFX-responders, compared to IFX naïve and IFX non-responders, even though we did not detected differences in the frequency of IL-10-producing Tregs among the groups of patients.

The discrepancy between branded and biosimilar infliximab may be due to various reasons. There was a trend towards an increase in the overall percentage of central and effector memory Th9 lymphocytes after exposure to biosimilar infliximab, but the difference was not significant, possibly because of the small number of patients. Furthermore, our experiments were carried using single batches of Remicade^®^ and Remsima^®^, but it is known that there may be structural differences between one batch and another of the same drug. The epitopes recognised by Th9 cells upon exposure to branded infliximab may not be the same as those recognised upon exposure to the biosimilar, due to differences in charge, amount of aggregates and unassembled forms, and post-translational motifs such as the pattern of glycosylation [28,29]. The greater fucosylation in the crystallisable fragment (Fc) of biosimilar infliximab may prevent interaction with the FcγR (especially FCγ RIIIa and FCγ RIIIb) of mononuclear cells [30,31]. This has been related to reduced antibody-dependent cell cytotoxicity (ADCC) and may also affect immunogenicity, by reducing the internalisation of the drug–receptor complex in antigen-presenting cells. It has also been demonstrated that the infliximab originator has at least two B cell epitopes in the Fc with a glycosylated pattern [32], and this may give it a different immunogenic profile from that of its biosimilar compound. Finally, although current randomised controlled trials and spontaneous reports have indicated comparable immunogenicity profiles between biosimilar and reference infliximab [33,34,35,36,37,38], these have all been based on the production of ADAs, which may depend on different immunogenic properties and biological pathways.

One of the main limitations of this study was that none of the biologically-treated patients (enrolled prior of the commercialization of biosimilar drugs) received a treatment with biosimilar infliximab, being all treated with Remicade^®^. Consequently, data on the association between the efficacy/safety profile of Remsima^®^ in vivo and Th9 cell percentages in vitro were not available. Moreover, differences in methotrexate and steroid intakes between the two groups (infliximab responders and non-responders) may have affected Th9 cell responses in vitro, despite no influence on Th9 cell percentages at baseline being reported and a lack of current scientific evidence.

## 4. Materials and Methods

### 4.1. Population

The study involved 55 outpatients with RA, diagnosed according to the ACR/EULAR 2010 criteria [39] who had participated in a previous study designed to explore the Th1/Th17-driven immunogenicity of infliximab (Remicade^®^) [13]: Fifteen subjects free of immunosuppressive drugs, 20 patients successfully treated with branded infliximab, and 20 patients who had switched or swapped from branded infliximab to other biological drugs because of adverse events or inefficacy. 

The patients and a matched control group of 10 healthy subjects were consecutively enrolled between June 2013 and December 2013.

The exclusion criteria were concurrent infections, atopic dermatitis, hematological disorders, concomitant or recent treatment with leflunomide or cyclosporine, or vaccinations in the previous two months, because these drugs or medical conditions can variously affect the Th cell pool [40,41,42,43,44].

The protocol was approved by the local Ethics Committee of the University Hospital Luigi Sacco, Milan, on 27 June 2013, registered with the number 364/2013 38AP (Resolution No. 484), and conduced in accordance with the Declaration of Helsinki. Written informed consent was obtained from all participants.

### 4.2. Immunological Analyses

Peripheral blood mononuclear cells (PBMCs) were isolated from 18 mL blood samples collected into EDTA-containing Vacutainer tubes (Becton Dickinson, Rutherford, NJ, USA) by means of centrifugation on lymphocyte separation medium (Cedarlane Laboratories, Burlington, NC, USA), and their number and viability were determined using an ADAM-MC automatic cell counter (Digital-Bio, NanoEnTek Inc., Seoul, South Korea). The cells were cultured in RPMI 1640 plus penicillin, streptomycin, L-glutamine and 10% pooled human AB serum (all from Euroclone, Siziano, Italy) at a concentration of 1 × 106/mL, and were incubated for 18 h with culture medium alone, branded infliximab 50 μg/mL (Remicade^®^, Janssen Biologics, Leiden, The Netherlands), or its biosimilar (Remsima^®^, Celltrion Healthcare, Budapest, Hungary); pokeweed mitogen (PWM) (1 μg/mL of lectin from Phytolacca Americana, Sigma-Aldrich, Saint Louis, MO, USA) was used as a positive control to evaluate the cells’ responsiveness [13]. The infliximab concentration of 50 μg/mL was chosen after titration testing—increasing drug concentrations and measuring median serum infliximab concentrations one hour after infusion (peak serum concentration: 39.9–219.1 μg) [45]. In order to facilitate co-stimulation, 1 μg/mL of anti-human CD28 (R&D Systems, Minneapolis, MN, USA) was added to the cell cultures. Brefeldin A 10 μg/mL (Sigma-Aldrich) was added after the first three h, in order to inhibit cytokine secretion.

The percentage of Th9 lymphocytes was determined by flow cytometric analysis. Th9 lymphocytes were identified as PU.1 and IRF4-expressing, IL-9-secreting CD4^+^ T cells, although the percentage of OX40-expressing, IL-9-secreting CD4^+^ T cells was also measured, as the co-stimulatory molecule may selectively drive Th differentiation toward a Th9 phenotype, while repressing both Treg and Th17 cell development [2]. IL-9 production by naïve CD4^+^ T lymphocytes (CCR7^+^, CD45RA^+^), central memory CD4^+^ T lymphocytes (CCR7^+^, CD45RA^−^), effector memory CD4^+^ T lymphocytes (CCR7^−^, CD45RA^−^), and terminally differentiated effector memory (TEMRA) CD4^+^ T lymphocytes (CCR7^−^, CD45RA^+^) was also evaluated.

The following human monoclonal antibodies (mAbs) were used: CD4 PE-Cy7, CD45RA FITC (Beckman Coulter, Milan, Italy); IRF4 PerCP-eFluor^®^ and CD134 (OX40) FITC (eBioscience, Diego, CA, USA); IL-9 allophycocyanin (APC), CCR7 R-phycoerythrin (PE) and PU.1 PE (R&D Systems). To evaluate the percentage of IL-9-secreting PU.1- and IRF4-expressing CD4^+^ T lymphocytes, the PBMCs were incubated with the mAbs for detecting cell surface antigens for 15 min, permeabilised with fixation/permeabilisation buffer (eBiosciences) for 30 min at 4 °C, and then stained with the antibodies for detecting intracellular transcription factors and IL-9 for a further 30 min at 4 °C. To evaluate IL-9 production by naïve and memory CD4^+^ T cells, the PBMCs were incubated with the mAbs for detecting cell surface antigens for 15 min at room temperature (RT), fixed with 1% paraformaldehyde (PFA) for 15 min at 4 °C, permeabilised with saponin (Sigma-Aldrich), and stained with the antibodies for detecting intracellular cytokines. Following 45 min of incubation in ice, the cells were fixed with 1% PFA.

The lymphocyte population was gated on the basis of its forward and side scatter properties, and further gated for CD4, CCR7 and CD45RA expression; at least 20,000 events were acquired within the CD4 gate. The samples were acquired using a Gallios flow cytometer, and the data were analysed using Kaluza software (both Beckman Coulter).

### 4.3. Statistical Analysis

The data were analysed parametrically, as they were normally distributed. The groups were compared using an unpaired Student’s *t* test for unequal variances, with a two-tailed *p* value and Pearson’s Chi squared test. A multivariate analysis was used to detect whether the subjects’ demographic characteristics or therapeutic regimens affected the Th9 percentages. A *p* value of <0.05 was considered statistically significant. The analyses were made using GraphPad Prism Software (GraphPad Software, San Diego, CA, USA) and SPSS, version 24.0 (International Business Machines Corporation, New York, NY, USA).

## 5. Conclusions

In conclusion, the prevalence of Th9 cells is higher in RA patients than in healthy subjects, and may be restored by concomitant conventional and biological treatments. Based on our findings, PU.1^+^, IRF4^+^ Th9 cells may be involved in orchestrating immune responses against epitopes of branded infliximab in patients failing the treatment; and this may be due to the recall and stimulation of both central and effector memory cells. On the other hand, despite a comparable profile in terms of immunogenicity emerged from other studies, biosimilar infliximab does not seem to activate these cell pools.

This study provides new insights into the immunogenicity of anti-TNF agents, which is routinely based on the detection of ADAs. The paradoxical activation of Th9 cells following exposure to infliximab may contribute to underlying inflammation, and thus explain the progressive loss of efficacy.

Nevertheless, the discrepancy between the Th9-driven immunogenicity of branded and biosimilar infliximab observed in our experiments may be attributable to our methodology rather than the real presence of dissimilar epitopes, and therefore deserves further investigation.

## Figures and Tables

**Table 1:** Demographic characteristics of the population included in the study. RA: rheumatoid arthritis; IFX: infliximab; SD: standard deviation; F: females; M: males; ACPAs: anti-citrullinated-protein antibodies; RF: rheumatoid factor; ANAs: anti-nuclear antibodies; anti-dsDNA: anti-double stranded DNA antibodies; anti-ENAs: anti-extractable nuclear antigen antibodies; ACLAs: anticardiolipin antibodies; LAC: lupus anticoagulant; CRP-DAS28: C-reactive protein/28-joint disease activity score; NSAIDs: non-steroidal anti-inflammatory drugs.

Variables	Healthy Controls	Treatment-Naïve RA Patients	RA Patients Responding to IFX	RA Patients Non-Responding to IFX
Number of subjects	10	15	20	20
Mean age ± SD, years	43.9 ± 8.3	54.8 ±16.2	61.3 ± 12.2	57.0 ± 12.2
Mean disease duration ± SD, years	/	2.3 ± 3.9	13.4 ± 7.2	18.1 ± 9.5
Gender, F/M (number)	4/6	12/3	16/4	15/5
ACPAs+, (number)	/	5	15	15
RF+, (number)	/	7	11	15
ANAs+, (number)	/	2	12	17
Anti-dsDNA Ab, (number)	/	0	3	2
Anti-ENAs Ab+, (number)	/	0	1	3
ACLAs/LAC+, (number)	/	0	1	2
Mean CRP-DAS28 ± SD	/	4.6 ± 1.0	2.5 ± 1.0	2.9 ± 0.8
Prednisone (2.5–10 mg/day), (number)	/	/	8	14
Methotrexate (5–15 mg/week), (number)	/	/	20	9
Hydroxychloroquine (200–400 mg/day), (number)	/	/	3	5
NSAIDs, (number)	/	14	as needed	as needed
